# A Comparison of the Effects of Packaging Containing Nano ZnO or Polylysine on the Microbial Purity and Texture of Cod (*Gadus morhua*) Fillets

**DOI:** 10.3390/nano8030158

**Published:** 2018-03-12

**Authors:** Małgorzata Mizielińska, Urszula Kowalska, Michał Jarosz, Patrycja Sumińska

**Affiliations:** Center of Bioimmobilisation and Innovative Packaging Materials, Faculty of Food Sciences and Fisheries, West Pomeranian University of Technology Szczecin, Janickiego 35, 71-270 Szczecin, Poland; urszula.kowalska@zut.edu.pl (U.K.); michal.jarosz@zut.edu.pl (M.J.); patrycja.suminska@zut.edu.pl (P.S.)

**Keywords:** cod fillets, *Gadus morhua*, ZnO nanoparticles, antimicrobial coatings, texture, active packaging

## Abstract

Portions of fresh Baltic cod fillets were packed into cellulose boxes (control samples), which were covered with Methyl Hydroxypropyl Celluloses (MHPC) coating with 2% polylysine. The cellulose boxes had square PE films and were enclosed in MHPC coating containing ZnO nanoparticles. The cod fillets were stored at 5 °C and examined after 72 h and 144 h storage times. Results obtained in this study showed that the textural parameters of the cod fillets increased, with both Springiness and Cohesiveness found greater after 144 h of storage for all analysed packaging materials. The Gumminess of fillets increased after storage, but the lowest increase was noted in cod samples that were stored in boxes containing PE films with ZnO nanoparticles. It was found that water loss from the cod fillets in these boxes was also lowest. The Adhesiveness of the fish samples stored in boxes devoid of active coatings also increased. In contrast to the packaging material devoid of active coatings, the storage of fillets in active coating boxes resulted in a decrease of adhesiveness. Microbial analysis showed that packaging material containing nano-ZnO was found to be more active against mesophilic and psychotropic bacterial cells than the coatings with polylysine after 72 h and 144 h of storage.

## 1. Introduction

Fish and fish products have high nutritional value and contain beneficial amounts of protein, lipids, essential minerals, and vitamins. However, fish are often considered to be difficult foodstuffs due spoilage and oxidation problems, as well as the development of off-flavours from improper handling or incorrect storage [1]. Fish spoilage is primarily caused by microbial growth and metabolism, and is characterized by changes in the sensory properties leading to unacceptable product quality. The shelf life of fish is affected by several factors, including storage temperature, fish species, initial microbial contamination and packaging conditions. Even though 10^7^ cfu/g is generally considered a maximum acceptable microbial load for fish, sensory rejection is typically found at microbial levels between 10^6^ and 10^9^ cfu/g [2]. The shelf life of fresh fish is generally limited by the growth of psychotropic Gram-negative rod-shaped bacteria along with Gram-positive microbes. In marine fish stored under refrigerated aerobic conditions, *Pseudomonas* sp. and *Shewanella* spp. have been observed to dominate [1,2]. As a result of microbial metabolism, odour is one of the most important quality determinants for fish freshness. Volatile organic compounds (VOCs), such as acids, alcohols, aldehydes, amines, ketones and sulphides, are often produced by bacteria leading to the production of characteristic off-odours and off-flavours [2]. Raw material quality and low storage temperature significantly reduce any deterioration in product quality, though time remains a highly limiting variable [3,4], with the preservation of the high nutritional quality of fish being of significant importance. Preservation methods that were studied in the 1980s sought to preserve fish and extend its shelf life using safe chemical preservatives such as potassium sorbate, shown to inhibit the bacteria responsible for spoilage odour. The addition of 2.5% and 5.0% of Potassium Sorbate to cod fillets, as well as packaging them in either 0.75 mm LDPE increased the shelf life of the fish by up to 16 days [5].

Active packaging is an innovative approach to maintain or prolong the shelf life of food products while ensuring their quality, safety and integrity [6,7]. Modified atmosphere packaging (MAP) can significantly prolong the shelf life of cod at chilled temperatures. Numerous studies have been carried out on the effects of MAP on the shelf life and quality retention of cod [8]. The reported shelf life of MA packed cod ranges from 10 to around 20 days at 0 to 3 °C [3,4,8]. Antimicrobial active packaging is a promising technology for the improvement of safety, and to delay spoilage during the processing and handling of fish. Applying antibacterial substances directly onto the surface of the fish has limited benefits, being neutralized on contact, or diffusing rapidly into the fish. The application of antimicrobial agents incorporated into a polymer matrix or their use as active coatings for covering packaging materials is a wide area of research for seafood packaging [1,6]. 

Recently, new types of nano-inorganic antimicrobial materials have become widely used in many fields, due to their stability at high temperatures and pressure conditions, they are generally considered safer for human and animals in contrast to organic substances. Antimicrobial polymers containing silver ions (Ag^+^) are preferred for their wide spectrum of antimicrobial activity, safety and heat stability [1]. Several researchers have blended PE with Ag, TiO_2_, and kaolin nanopowders for the preservation of fresh food stored at 4 °C for 12 days. Zinc Oxide (ZnO) nanoparticles have also been explored as an antimicrobial agent, used in active food packaging systems. These nanoparticles are recognised as safe (GRAS) by the United States food and drug administration (USFDA, 21CFR182.8991) [9,10,11]. Zinc Oxide nanoparticles offer bactericidal effects for Gram-positive and Gram-negative bacteria and to spores that are resistant to high temperature and high pressure, as well as yeasts and moulds [11]. Zinc Oxide nanoparticles have been incorporated into polymers and have been added to biodegradable active coatings [7,11,12,13,14]. Numerous studies have shown an increase in the shelf life of food products packed in films containing ZnO nanoparticles (within a polymer matrix or added into an active coating) [15,16,17,18]. The shelf life of sliced wheat bread was extended from 3 to 35 days using packaging containing nanoparticles when compared to control versions [15]. All active coatings reduced the number of microorganisms in sliced bread for up to 15 days. Films containing nano-ZnO exhibited excellent antimicrobial activity and were fabricated into packaging pouches for raw meat. The prepared pouches showed significant action against bacteria in the meat, offering complete inhibition of microbial growth for to six days of storage at 4 °C [4]. Emamifar et al. [19] reported that LDPE nanocomposite packaging materials containing Ag and ZnO nanoparticles were conducive in prolonging the shelf-life of fresh orange juice in storage at 4 °C. Li et al. [20,21,22] successfully developed a packaging material containing nano-ZnO particles as active food packaging to improve the shelf-life of freshly cut apple. However, one shortcoming relating to the use of nanoparticles in food packaging is the migration of nanoparticles from the packaging materials to the food, which can harm human health and have a negative effect on environmental safety. Li et al. [21,22] confirmed that the amounts of nanoparticle migration from the nano-blend film to cheese samples and food simulants were far below the migration limit of 1 mg/kg as defined by EFSA for food contact materials. ε-Polylysine (PL) as a natural antimicrobial polypeptide is also recognized as safe and the antimicrobial action of PL is attributed to its polycationic and surface nature that enables its interaction with bacterial membranes. The polypeptide is active against G(+) and G(−) food pathogenic bacteria including *Listeria monocytogenes*, *Escherichia coli* O157:H7 and *Salmonella Typhimurium*. However, studies in the literature related to use of PL in antimicrobial packaging are scarce. Zinoviadou [23] is noted as the first researcher using PL in whey protein films and successfully applying the developed films to control the spoilage flora of fresh beef. Ünalan [24] analysed the antimicrobial properties of edible films from whey proteins, alginate, zein and chitosan incorporated with polylysine. PL is also used in Japan as an antimicrobial preservative in foods. Different Japanese foods that contain PL include sliced fish and fish surimi, boiled rice, noodle soup stocks, noodles and cooked vegetables [24].

The purpose of this research was to compare the effect of packaging containing nano ZnO or polylysine on microbial purity and cod fillet texture.

## 2. Materials and Methods

Fresh Baltic cod fillets *(Gadus morhua callarias)* (Świeża-Ryba.pl, Szczecin, Poland) were ordered online (Świeża-Ryba.pl) and brought (in polystyrene (PS) boxes containing ice) to the Center of Bioimmobilisation and Innovative Packaging Materials (CBIMO). 

Cellophane/Biopolyethylene films (Cel/PE, (A4, 50 μm) (Be Nature, Schoten, Belgium) were used in this research. Methyl Hydroxypropyl Cellulose (MHPC, Chempur, Piekary Śląskie, Poland) and Methyl Cellulose (Methocel™, Dow, Stade, Germany) were used as coating carriers. Zinc Oxide AA 44,899, (~70 nm) and polylysine (Handary, Uccle, Belgium) were used as active substances. To verify the antimicrobial purity of the cod fillets, PPS, PCA and MRS mediums (Biocorp, Warsaw, Poland) were used. The mediums were prepared according to the Biocorp protocol (all mediums were weighed according to the manufacturer’s instructions, then suspended in 1000 mL of distilled water and autoclaved at 121 °C for 15 min).

Cellulose boxes (Celabor, Herve, Belgium) and PE films were used as packaging materials for the fresh cod fillets.

### 2.1. Coatings Preparation

(1) Two grams of Methocel™ were introduced into 96 mL of water. The mixture was mixed for 1 h using a magnetic stirrer (Ika) at 1500 rpm. Next, 2 g of polylysine were introduced into 98 g of mixture. The mixture was then mixed for 1 h using a magnetic stirrer (Ika) at 1500 rpm. The mixture was used to cover the cellulose boxes to obtain 2% polylysine coatings as active substance.
(2) Exactly 0.082 g of ZnO nanoparticles were introduced into 50 mL of water. Initially, the mixture was mixed for 1 h using a magnetic stirrer (450 rpm). Next, the mixture was sonicated (sonication parameters: cycle: 0.5; amplitude: 20%; time: 10 min), while, at the same time, a second mixture (4 g of MHPC into 50 mL) was prepared as described above. The ZnO nanoparticles solution was introduced into the MHPC mixture and sonicated (sonication parameters: cycle: 0.5; amplitude: 20%; time: 10 min).

Polyethylene (PE) films (20 μm, CBIMO—Center of Bioimmobilisation and Innovative Packaging Materials, Szczecin, Poland) were covered using Unicoater 409 (Erichsen, Hemer, Germany) at 25 °C with a roller at a diameter of 40 μm. The coatings were dried for 10 min at a temperature of 50 °C. Finally, 1.6 g layers of MHPC per 1 m^2^ of PE were obtained. The active coatings contained 0.032 g of ZnO AA 44,899 particles per 1 m^2^ of PE film. 

The covered film samples were cut into square shapes (9 cm × 9 cm) and introduced into the cellulose boxes. 

### 2.2. Packaging and Storage

**Figure 1:**
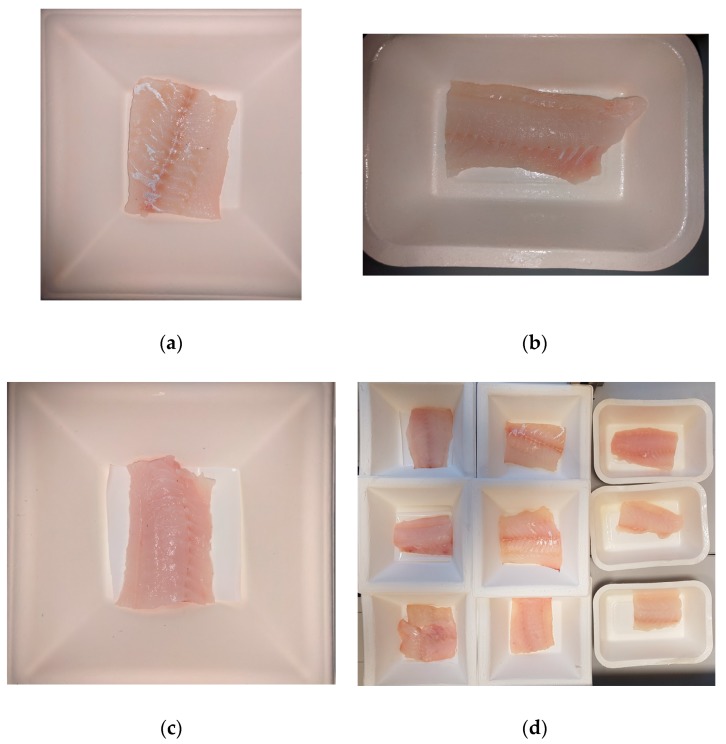
(**a**) The cod in the control box; (**b**) the cod in the box covered with a coating with polylysine; (**c**) the cod in the box covered with a coating with ZnO nanoparticles; and (**d**) the cod before welding.

The fresh Baltic cod fillets were cut into 25 g pieces. The cod portions were packed into cellulose boxes that were welded with Cel/PE films. The samples were aseptically introduced into:

a. Cellulose boxes (control samples) (Figure 1a,d);
b. Cellulose boxes covered with a Methocel™ coating with 2% polylysine (Figure 1b,d); and
c. Cellulose boxes with square PE films covered with MHPC coating containing ZnO nanoparticles (Figure 1c,d).

The fillets were put on the film squares and covered with the PE square films. The cod fillets were in contact with the active coatings on both sides. 

Next, the boxes were joined with Cel/PE films using a welder (HSE-3, RDM Test Equipment, Hertfordshire, UK) in normal air conditions. The parameters of welding were: Temperature, 145 °C (box covered with coating) and 150 °C (box devoid of coating); Pressure, 3 kN; and time, 1 s. 

The boxes containing cod fillets were then stored in a refrigerator. The samples were stored at 5 °C. The cod fillets were examined after 72 h and 144 h of storage.

### 2.3. Mechanical Analysis

The texture analysis of the cod fillets was carried out according to the PN-ISO 11036:1999 standard: “Sensory analysis. Methodology. Texture profiling” [25]. The tests were carried out using Zwick/Roell Z 2.5 (Wrocław, Poland).

### 2.4. Microbiological Purity

For microbiological analysis, 25 ± 0.1 g of individual fillet was aseptically introduced into a sterile stomacher bag and in physiological saline peptone solution (PPS: 0.85% *m/v* NaCl, 0.1% *m/v* peptone). The samples were homogenized in a Bag Mixer (Interscience, Saint-Nom-la-Brèteche, France) for one minute and appropriate decimal dilutions were prepared in PPS. The total psychrotrophic count (TPC) and total were determined according to PN-EN ISO 4833-2:2013-12 [26]; PN-ISO 17410:2004 [27] and PN-EN ISO 6887-3:2017-05 [28] standards.

### 2.5. Dry Mass Tests

Dry mass was measured for fresh cod (control sample) before being added into boxes after 72 h and 144 h of storage. Dry mass analysis was performed using a Weight Dryer (Radwag, Warsaw, Poland). The test was performed in duplicate. 

### 2.6. L* a* b* Tests

Product colour was determined as an average of 9 measurements from randomly selected fillet spots with a colometer (NR 20XE, EnviSense) and related data software. Colour was measured through an aperture (a diameter 8 mm) using the CIE L* a* b* colour space with a standard 10 observer and Illuminant D65. The selected parameters (to describe the results) were ∆*E_lab_* (total colour aberration) and ∆*L* (the difference between lightness and darkness). The parameters were calculated according to an EnviSense protocol. 

### 2.7. Statistical Analysis

Statistical significance was determined using an analysis of variance (ANOVA) followed by a Duncan test. The values were considered as significantly different when *p* < 0.05. All analyses were performed with Statistica version 10 (StatSoft Polska, Kraków, Poland).

## 3. Results 

### 3.1. Microbial Purity Analysis

**Figure 2:**
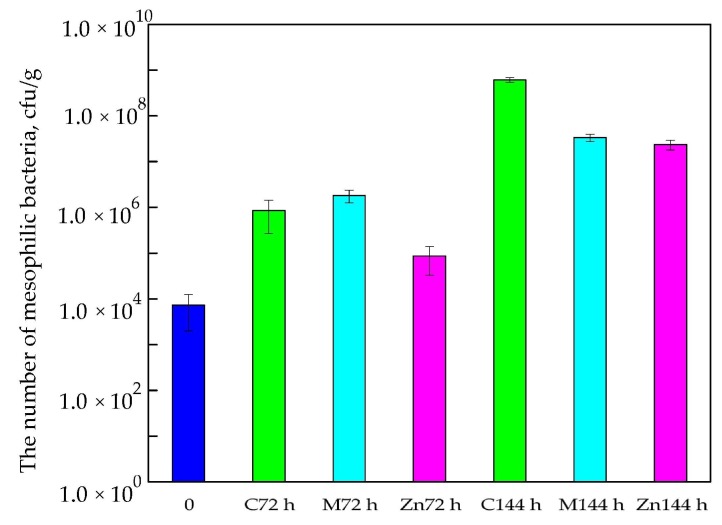
The number mesophilic bacteria after 72 h and 144 h of storage.

Results of the study demonstrated that the amount of mesophilic bacterial cells from cod fillets stored in boxes devoid of active coatings or active films (C—control sample) increased after 72 h of storage at 5 °C in air conditions. A 2-log increase in the number of bacteria was observed (compared to the “0” sample—before storage). Figure 2 shows that a Methocel™ coating containing 2% polylysine had no influence on the growth of microorganisms. A 2-log increase in the number of living cells was also observed (than compared to the “0” sample—before storage). It is tempting to suggest that the coating was not active against mesophilic bacteria. As emphasized below (Figure 2), the PE films covered with MHPC coating containing ZnO nanoparticles (introduced into boxes) decreased the number of mesophilic bacteria compared to the boxes devoid of coatings or covered with Methocel™ with 2% polylysine. A 1-log increase in the number of bacteria was noted (than compared to the “0” sample—before storage). Statistical analysis demonstrated that any differences between the numbers of microorganisms were not significant (*p* > 0.05).

As observed in this study, different results were obtained for cod fillets after 144 h storage at 5 °C in air conditions. A comparison of “0” samples (the number of bacteria isolated from cod fillets before storage) and control samples (the number of bacteria isolated from cod fillets stored in boxes devoid of active coatings or active films) showed that the amount of mesophilic bacteria increased significantly. A 5-log increase in the number of microorganisms was observed compared to the “0” samples and a 3-log increase in the number of bacteria was observed compared to control samples stored for 72 h. The differences between the numbers of viable cells were significant, which was confirmed by a Duncan test (*p* < 0.05).

It was noted that active coatings with 2% polylysine covering the boxes and active coatings with ZnO nanoparticles that covered the films were active against mesophilic bacteria (for cod samples stored for 144 h). A 4-log increase in the number of microorganisms was observed compared to the “0” samples and a 2-log increase in the number of bacteria was observed compared to control samples stored for 72 h. The differences between the numbers of viable cells were significant, as confirmed by a Duncan test (*p* < 0.05). It should be mentioned that 10^7^ cfu/g is generally considered a maximum acceptable microbial load for fish. This means that the cod fillet shelf life stored in boxes devoid of active coatings or active films should have been less than 144 h, as the number of mesophilic bacteria for these samples was 6.15 × 10^8^ cfu/g, a higher number than the acceptable microbial load for cod that could be consumed. A previous study [11,13] confirmed that MHPC coatings containing ZnO nanoparticles inhibited the growth of Gram-positive and Gram-negative bacteria. The results of that study demonstrated that MHPC coatings with nano-ZnO were more active against mesophilic bacterial cells than coatings with polylysine as an active substance after 72 h and 144 h of storage. 

**Figure 3:**
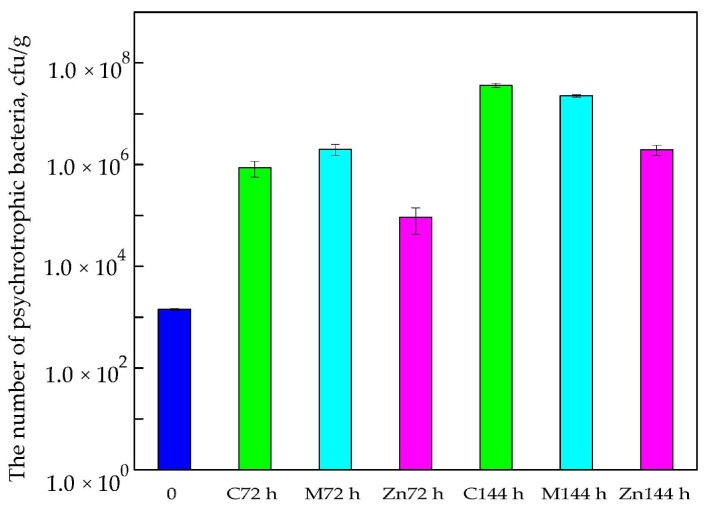
The number psychotropic bacteria after 72 h and 144 h of storage.

Results from our study showed that the amount of psychotropic bacteria isolated from cod fillets stored in boxes devoid of active coatings or active films (C—control sample) increased after 72 h of storage at 5 °C in air conditions. A 2-log increase in the number of bacteria was observed (than compared to the “0” sample). It was noted that boxes covered with a Methocel™ coating containing 2% polylysine were found to be the worst solution. A 3-log increase in the number of living cells was observed in these samples (than compared to “0”). It is tempting to suggest that the coatings must have been used as an additional carbon source by psychrotrophic bacteria. As emphasized below (Figure 3), the PE films covered with MHPC coating containing ZnO nanoparticles reduced the number of psychotropic bacteria compared to boxes devoid of coatings or covered with Methocel™ with 2% polylysine. A 1-log increase in the number of bacteria was noted (than compared to the “0” sample). A statistical analysis demonstrated that the differences between the numbers of microorganisms were significant (*p* < 0.05).

It was observed in this study that cod fillets could be stored for 144 h in 5 °C air conditions. A comparison of “0” samples and control samples showed that the amount of psychotropic bacteria increased significantly. A 4-log increase in the number of microorganisms was observed compared to “0” samples and control samples stored for 72 h. The differences between the numbers of viable cells were significant, which was confirmed by a Duncan test (*p* < 0.05).

It was observed that active coating ZnO nanoparticles covering the films were active against psychotropic bacteria (for cod samples stored for 144 h). A 3-log increase in the number of microorganisms was observed compared to the “0” samples and a 1-log increase in the number of bacteria was observed than when compared to the control samples and M samples stored for 72 h. The differences between the numbers of viable cells were significant, and this was confirmed by a Duncan test (*p* < 0.05). It should be mentioned that the number of microorganisms in cod fillets stored in boxes containing ZnO nanoparticles was 10^6^ cfu/g. This suggests that the shelf life of the cod fillets packed in these boxes could be greater than 144 h. The results of the study showed that the MHPC coatings with nano-ZnO were more active against psychotropic bacterial cells than coatings with polylysine as an active substance after 72 h and 144 h storage. 

### 3.2. Mechanical Analysis

**Figure 4:**
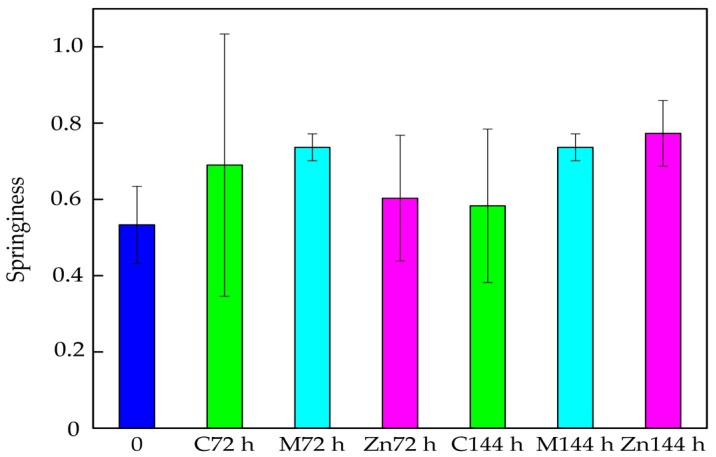
The Springiness of the cod fillets after 72 and 144 h of storage.

The results of the study demonstrated that the Springiness of the cod fillets increased after 72 h of storage in boxes devoid of active coatings or active films. After 144 h this parameter decreased. A modification of the boxes with active coatings containing polylysine as an active substance caused a greater increase in the cod fillet Springiness after 72 h and 144 h storage (Figure 4). The differences between Springiness values were found to not be significant, and this was confirmed by statistical analysis (*p* > 0.05). An increase in the Springiness of the cod fillets stored in boxes containing ZnO nanoparticles after 72 h and 144 h was also observed.

**Figure 5:**
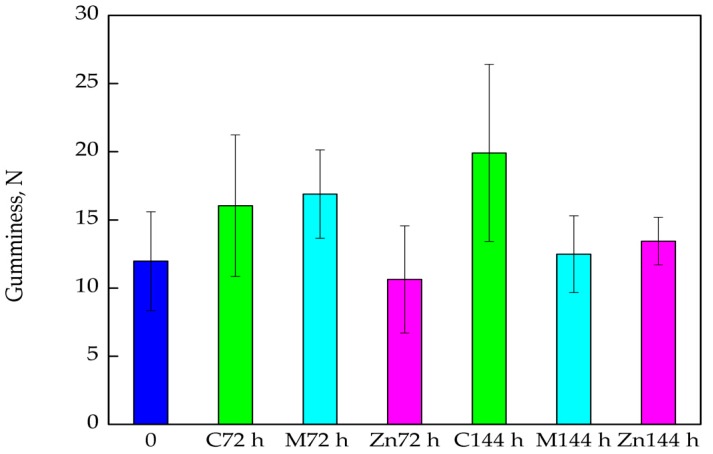
The Gumminess of the cod fillets after 72 and 144 h of storage.

As emphasized below (Figure 5), the Gumminess of cod samples stored in boxes devoid of active films or coatings (control sample) increased after 72 h and 144 h than compared to the “0” sample. These changes were significant and were confirmed by Duncan test (*p* < 0.05). Similarly, Methocel™ coatings with polylysine caused a significant increase in this parameter after 72 h of storage, again, confirmed by a Duncan test (*p* < 0.05). After 144 h of storage, the average Gumminess value of the cod fillets stored in boxes covered with Methocel™ containing polylysine, decreased when compared to the Gumminess value of the samples stored for 72 h, and increased compared to the “0” sample. The differences between Gumminess values were found to not be significant, which was confirmed by statistical analysis (*p* > 0.05). Additionally, after 72 h and 144 h storage, the Gumminess of the cod stored in boxes with PE films covered with MHPC coatings containing ZnO nanoparticles showed a slight increase compared to the Gumminess value obtained from the “0” sample. The differences between Gumminess values were not significant, which was confirmed by Duncan test (*p* > 0.05).

**Figure 6:**
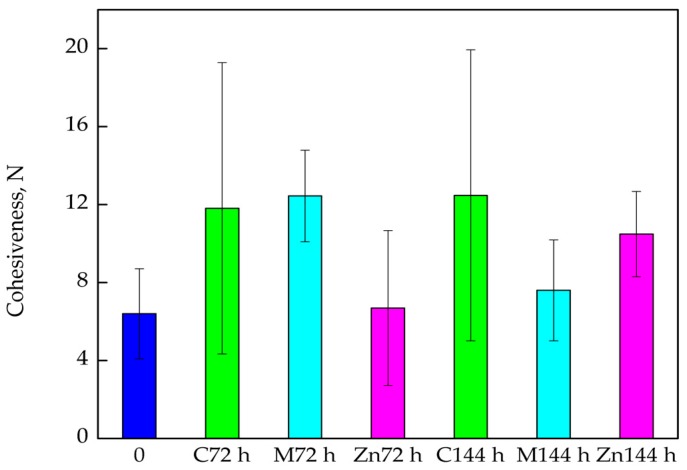
The Cohesiveness of the cod fillets after 72 and 144 h of storage.

In the case of cohesiveness, it was observed that the average parameter value for the fillets stored in boxes prepared as control samples increased after 72 h storage (Figure 6). These changes were significant and confirmed by Duncan test (*p* < 0.05). The average Cohesiveness value, measured for the same samples, but stored for 144 h also increased, although the amount was small. The differences between Cohesiveness values were found to not be significant, again confirmed by statistical analysis (*p* > 0.05). A Significant increase in this parameter was observed for samples stored for 72 h in coated boxes (control samples) (*p* < 0.05). After 144 h storage, a decrease in cod Cohesiveness was observed, but the differences were found to not be significant (*p* > 0.05). An increase in fillet Cohesiveness was also observed for samples stored for 72 h and 144 h in packaging containing ZnO nanoparticles. It should be mentioned that the increase was not significant, again confirmed by Duncan test (*p* > 0.05).

**Figure 7:**
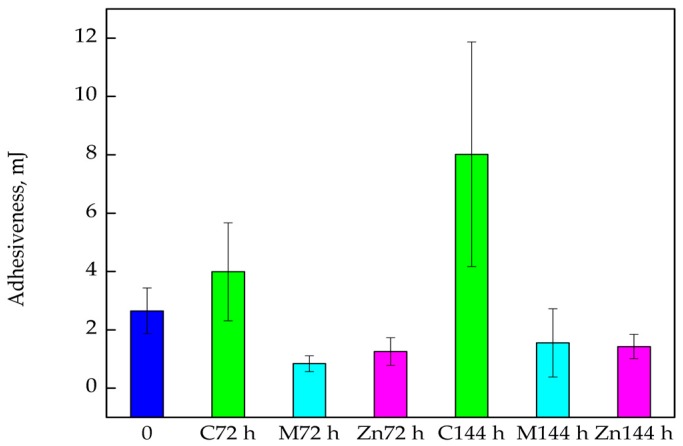
The Adhesiveness of the cod fillets after 72 and 144 h of storage.

The results showed that Adhesiveness was a parameter that clearly depended on the packaging material (Figure 7). Analysing the Adhesiveness values of the cod fillets that were introduced into boxes devoid of active coatings or covered PE films for 72 h, it was noted that this parameter increased, though not significantly (*p* > 0.05). After 144 h storage an increase was found to be significant and this was confirmed by Duncan test (*p* < 0.05). Adhesiveness of all cod samples that were introduced into the boxes that were covered with Methocel™ with polylysine or into the boxes containing PE films covered with MHPC with ZnO nanoparticles, decreased after 72 h and after 144 h storage. Results showed that the modification of boxes with an active coating containing or introducing active films into the boxes changed Adhesiveness significantly, which was confirmed by statistical analysis (*p* < 0.05). 

### 3.3. Dry Mass Analysis

The results of this study demonstrated that the dry mass of fresh cod fillets was 17.64%. The storage of fillets in cellulose boxes led to an increase in the dry mass of cod to 20.00% after 72 h storage and to 23.06% after 144 h. It was noted that the dry mass of fillets stored in boxes covered with active coatings was higher than the dry mass of the samples stored in boxes devoid of active coating. Nanoparticles of ZnO led to a decrease in the dry mass of the fish fillets. Water loss was the lowest for the samples stored in boxes containing PE films covered in coatings with ZnO nanoparticles. The highest water loss was obtained in cod fillets stored in boxes covered with Methocel™ containing polylysine (Table 1).

### 3.4. L* a* b* Analysis

It was determined in this study that ∆*E_lab_* depended on the packaging material (Table 2). ∆*E_lab_* of cod fillets that were introduced into the boxes devoid of active coatings or covered PE films for 72 h was lower than samples introduced into the boxes containing films with ZnO nanoparticles. In contrast to films coated with MHPC containing nano ZnO, ∆*E_lab_* for samples stored in boxes covered with Methocel™, with polylysine was higher. Different results were obtained after 144 h storage. The highest ∆*E_lab_* was obtained for samples stored in packaging coated with an active coating. The lowest ∆*E_lab_* was observed in the control sample. It was also noted that ∆L was also dependent on packaging material. An analysis of cod fillets stored for 72 h and 144 h, found that the highest ∆*L* values were obtained for samples introduced into boxes with films covered with coatings containing ZnO nanoparticles, and the lowest for boxes coated with Methocel™ with polylysine.

## 4. Discussion

Freshness is one of the most significant properties in the evaluation of fish quality, as this characteristic is directly linked to microbial purity, texture, and perception taste for consumers. Generally, fish is processed and frozen to guarantee palatability and safety, to improve shelf life and convenience, and to maintain and prolong freshness. The preservation of fresh cod fillets is problematic because fresh fillets undergo rapid deteriorative processes that can promote fish spoiling. At the same time, due to microbial growth, enzymatic degradation and cod fillet texture, they generally have a short shelf life [29,30,31]. The main mission of food packaging is to maintain the quality and safety of food products during storage and to extend shelf-life by avoiding unpleasant effects, such as hazardous microorganisms and their corresponding toxins, external physical force, chemical compounds, sunlight, permeable volatile compounds, oxygen and moisture [6,32]. Ünalan et al. [24] analysed the antimicrobial activity of edible films from whey proteins, alginate, zein and chitosan incorporated with polylysine and found polylysine to be effective against microorganisms. Numerous studies have also found ZnO nanoparticles to be active against microorganisms, and have shown an increase in the shelf life of food products packed in films containing incorporated ZnO nanoparticles (within a polymer matrix) or used with coatings containing ZnO nanoparticles [9,10,11,12,13,14,15,16,17,18,19,20]. 

One of the most important parameters determining the quality of fish fillets consumed without thermal processing is their texture and colour. This refers mainly to fish that are characterized by subtle flavour. A product that is too soft or not cohesive enough may cause problems when sliced or create doubts in the consumer as to its freshness. Cohesiveness is highly important parameter that represents forces holding the product together. On the other hand, tough, stringy or gummy products are not acceptable since they present non-specific and excessively strong resistance during mastication [33,34]. The results obtained in this study showed that the textural parameters of cod fillets after 144 h storage in relation to Springiness and Cohesiveness increased for all analysed packaging materials (when compared to the “0” sample). It is tempting to suggest that the storage of cod fillets in boxes improved the texture of fish, also backed up by Michalczyk and Surówka [33] in their results. The authors confirmed that textural parameters tended to increase during storage. The present study showed that, unfortunately, fillet Gumminess increased after storage and is considered a clear disadvantage. The best results were obtained for samples that were stored in boxes containing PE films with ZnO nanoparticles when the smallest increase of Gumminess in these samples was noted. It is also worth considering another advantage of the packaging material with ZnO nanoparticles: it was also shown that water loss in cod fillets from these boxes was the lowest. It was also observed that the highest ∆L values were obtained for the fillet samples introduced into boxes with films containing ZnO nanoparticles. This means that the cod fillets taken from these kinds of packaging were the lightest. 

The Adhesiveness of cod fillets stored in boxes devoid of active coatings also increased, contrary to packaging material devoid of active coatings, the storage of fillets in boxes with active coatings caused a decrease in adhesiveness. It should be mentioned that a greater decrease in this parameter was observed for coatings containing ZnO nanoparticles. The opposite results were observed by Ayala et al. [35], who analysed the textural parameters of fish fillets packed in air or vacuum conditions (into packaging devoid of any active coatings) and stored for 22 days. The authors found that the textural parameters decreased significantly with storage time. 

The results of this study demonstrated that MHPC coatings with nano-ZnO were more active against mesophilic and psychotropic bacterial cells than coatings with polylysine as an active substance after 72 h and 144 h storage. The ZnO nanoparticles decreased the number of microorganisms due to their greater activity against Gram-positive and Gram-negative bacteria presented in the previous study [11,13]. It is known that 10^7^ cfu/g is considered a maximum acceptable number of living microbial cells for fish. This means that the shelf life of cod fillets stored in boxes devoid of active coatings should be shorter than 144 h, as the number of mesophilic bacteria for these fillets was 6.15 × 10^8^ cfu/g. The number of the bacteria after 72 h of storage was 8.51 × 10^5^ cfu/g, which means that the cod fillets could be stored for 72 h in boxes devoid of active coatings. Quite contradictory results were obtained by Kuuliala et al. [2] who established that a limit of 7-log cfu/g isolated from cod samples was exceeded after two days of storage in air conditions at 4 °C. The authors contributed intelligent packaging technologies by identifying and quantifying volatile organic compounds (VOC) that indicated spoilage of raw *Gadus morhua* under MAP and air. They observed that 7-log cfu/g was needed for the onset of exponential VOC increase. When the fish was stored under air condition, *Pseudomonas* and *Shewanella* spp. were isolated. These microorganisms have been considered as Specific spoilage organisms (SSOs) of refrigerated or iced marine fish. The authors proved that a high carbon dioxide concentration in MAP packaging inhibited pseudomonads. Sivertsvik et al. [4] noticed that the raw cod fillets may be stored even 14 days, when MA packaging is applied and when the temperature is 0 °C. Typically, MA packaging achieves an increase of acceptable shelf life of about 50%, but it depends on the storage temperature, raw material quality, handling, and gas mixture ratio; and packaging material. The authors demonstrated that the optimum gas mixture ratio was 63 mL/100 mL O_2_ and 37 mL/100 mL CO_2_. It should be mentioned that shelf life of cod under MA-conditions has been reported from 10 to about 20 days at chilled (0–3 °C) or superchilled (−0.9 °C) temperatures [2,3,8].

The results of this research work demonstrated that the number of bacterial cells stored in boxes covered with Methocel™ containing polylysine or in boxes containing the PE films coated with MHPC with ZnO nanoparticles did not go over 10^7^ cfu/g. This suggests that the active coatings improved the quality of cod fillets after storage. It should also be added that coatings with nano ZnO were more active than coatings with polylysine. Summarizing, it should be mentioned that the boxes containing PE films with ZnO nanoparticles extended the quality and freshness of the cod fillets after 144 h storage at 5 °C under air condition. Similar results were obtained by Singh S. et al. [1] who applied the boxes containing polypropylene films with incorporated AgSiO_2_ which has both antimicrobial and amine-absorbing properties. The authors studied effect of PP + AgSiO_2_ composite (5.0 or 10.0%) on the microbiological, chemical, and colour properties of fresh fish in chilled storage (2 ± 0.5 °C). The authors indicated that active packaging may lead to the retention of quality, and extension of shelf life of fish, during refrigerated storage for up to seven days. In addition, the packages may also reduce the amount of odour-producing trimethylamine. 

## 5. Conclusions

The boxes containing PE films covered with MHPC with ZnO nanoparticles were found to be the best packaging material to extend the quality and freshness of cod fillets after 144 h storage at 5 °C. 

## Figures and Tables

**Table 1:** The dry mass of cod fillets after 72 h and 144 h of storage.

Time (h)	Dry Mass (%)			
	0	C	M	Zn
0	17.64	-	-	-
72	-	20.00	22.58	19.18
144	-	23.06	24.96	18.90

**Table 2:** The changes of colour of cod fillets after 72 h and 144 h of storage.

Time (h)		C	M	Zn
72	∆*E_lab_*	5.45 ± 3.00	3.70 ± 1.10	18.23 ± 0.26
	∆*L*	0.4 ± 6.15	−0.42 ± 0.83	17.91 ± 0.44
144	∆*E_lab_*	12.57 ± 0.70	54.03 ± 18.40	21.05 ± 5.10
	∆*L*	11.51 ± 0.74	0.49 ± 1.98	20.84 ± 5.04
